# Serological proteome analysis approach-based identification of ENO1 as a tumor-associated antigen and its autoantibody could enhance the sensitivity of CEA and CYFRA 21-1 in the detection of non-small cell lung cancer

**DOI:** 10.18632/oncotarget.17067

**Published:** 2017-04-12

**Authors:** Liping Dai, Yanhong Qu, Jitian Li, Xiao Wang, Kaijuan Wang, Peng Wang, Bing-Hua Jiang, Jianying Zhang

**Affiliations:** ^1^ Institute of Medical and Pharmaceutical Sciences, Zhengzhou University, Zhengzhou, Henan, 450052, China; ^2^ Department of Biological Sciences, The University of Texas at El Paso, El Paso, TX, 79968, USA; ^3^ Henan Key Laboratory for Tumor Epidemiology, Zhengzhou University, Zhengzhou, Henan, 450052, China; ^4^ Department of Epidemiology, College of Public Health, Zhengzhou University, Zhengzhou, Henan, 450052, China; ^5^ The Third Affiliated Hospital, Zhengzhou University, Zhengzhou, Henan, 450052, China; ^6^ Center for Molecular Carcinogenesis, Department of Pathology, Anatomy and Cell Biology, Thomas Jefferson University, Philadelphia, PA, 19107, USA

**Keywords:** alpha-enolase (ENO1), non-small cell lung cancer (NSCLC), tumor associated-antigens (TAAs), autoantibody, serological proteome analysis (SERPA)

## Abstract

**Purpose:**

Lung cancer (LC) is the leading cause of cancer-related deaths for both male and female worldwide. Early detection of LC could improve five-year survival rate up to 48.8% compared to 3.3% of late/distant stage. Autoantibodies to tumor-associated antigens (TAAs) have been described as being present before clinical symptoms in lung and other cancers. We aimed to identify more TAAs to improve the performance for discovering non-small cell lung cancer (NSCLC) patients from healthy individuals.

**Methods:**

Two independent sets were included in this study. Serological proteome analysis (SERPA) was used to identify TAAs from NSCLC cell line H1299 in a discovery set. In validation study, anti-ENO1 autoantibody was examined by immunoassay in sera from 242 patients with NSCLC and 270 normal individuals.

**Results:**

A 47 KDa protein was identified to be alpha-enolase (ENO1) by using SERPA. Analysis of sera from 512 participants by ELISA showed significantly higher frequency of anti-ENO1 autoantibodies in NSCLC sera compared with the sera from normal individuals, with AUC (95%CI) of 0.589 (0.539-0.638, *P*=0.001). There was no significant difference in frequency of anti-ENO1 in different stages, histological or metastasis status of NSCLC. When anti-ENO1 detection was combined with other two tumor protein biomarkers (CEA and CYFRA 21-1), the sensitivity of NSCLC increased to 84%.

**Conclusions:**

ENO1 can elicit humoral immune response in NSCLC and its autoantibody has association with the tumorigenesis of NSCLC. Furthermore, these intriguing results suggest the possibility of autoantibody against ENO1 serving as a potential diagnostic biomarker in NSCLC and have implications for defining novel histological determinants of NSCLC.

## INTRODUCTION

Lung cancer (LC) is the leading cause of cancer-related deaths for both male and female worldwide. In 2015, it was estimated more than 220,000 would be diagnosed as LC and 158,000 would die from LC [1]. Until 2007, the five-year overall survival rate for LC patients is only 16% among all cancers [2]. Early detection of LC could improve five-year survival rate up to 48.8% compared to 3.3% of late/distant stage [3]. Currently, low dose spiral computed tomography (LDCT) is the limited approach to screen LC in at-risk individuals in early detection of LC [4, 5]. LDCT offers mortality benefit in high risk individuals [6], nevertheless, this technique has poor specificity, needs plenty of costs [7], requiring individuals to have unnecessary follow-up examinations and unnecessary surgery therapy [8]. Therefore, the identification and validation of a cost-effective early stage blood test to complement LDCT screening is essential. Serum biomarker detection, which possesses advantages such as easy operation, low cost, noninvasiveness, accessibility of samples, is a high-profile topic for detection of early LC [9]. An growing number of studies have demonstrated autoantibodies to tumor-associated antigens (TAAs), and these humoral autoimmune responses have been described as being present before clinical symptoms in lung and other cancers [10–13]. We have recently reported that elevated autoantibody levels could be detected in patients’ sera at least four years before the diagnosis of LC [13].

In the present study, we investigated the possibility of defining novel TAAs by serological proteome analysis (SERPA), which could improve the performance characteristics for discovering the NSCLC patients from the healthy individuals. To achieve this goal, we used a differential immunoproteomic strategy based on two-dimensional Gel Electrophoresis (2-DE) coupled with mass spectrometry to identify TAAs in whole cell lysates prepared from the non-small cell carcinoma (NSCLC) cell line H1299 and sera pools from NSCLC patients at early stage as well as normal human individuals. The identified TAAs were subsequently validated using NSCLC patients in different stages and normal individuals to determine whether they are specific markers to differentiate NSCLC from normal population.

## RESULTS

### A 47 KDa autoantigen was identified as ENO1 by serum autoantibody from patients with NSCLC at early stage

**Figure 1:**
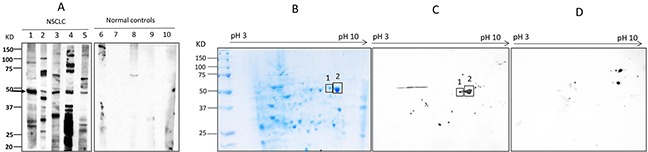
Western blotting analysis **(A)** Western blotting of representative serum samples with H1299 lung cancer cell line. Lanes 1-5 are lung cancer sera showing the common reactive bands with 47 KDa molecular weight, while lanes 6-10 (normal controls) are not observed corresponding bands in the same size. **(B-C)** Identification of the 47 KDa lung cancer protein by immunoproteomics. (B): 2-DE protein profile of H1299 cells. (C): 2-DE Western blotting analysis was probed with five lung cancer sera pool which contain antibodies to the 47 KDa protein. **(D)** 2-DE Western blotting of five normal human sera.

The objective of this study was to identify the specific autoantibodies and targeted antigens as biomarkers in lung cancer. An immunoproteomic approach known as SERPA has been well established in our lab to identify TAAs as biomarkers in cancers in our previous studies [14, 15]. The advantage of this method is based on the accurate identification of protein by mass spectrometry analysis. In initial study, 20 sera from patients with NSCLC at stage I and 20 matched normal individuals in discovery set were screened for the presence of autoantibodies against total protein extractions from NSCLC cell line H1299 by using Western blotting. Of interest, 5 of 20 (25%) NSCLC sera were observed containing antibodies against protein bands around 47 KDa. As shown in Figure 1A, no reactivity with the 47 KDa protein was detected in 20 normal human sera. Subsequently, proteins extracted from H1299 cells were separated by 2-DE (Figure 1B) and transferred onto the nitrocellulose membranes. Figure 1C shows two positive protein spots around 47 KDa after reacted with sera from NSCLC patients, while no corresponding spots were observed in Western blotting results for normal sera (Figure 1D). These two spots were cut out and analyzed by LC-MS/MS analysis. As shown in Table 1, proteomic analysis demonstrated that these two reactive protein spots matched with alpha-enolase (ENO1).

**Table 1:** Mass spectrum result for 47 KDa protein

No. in gel	Identified protein	Official Symbol	score	expected MW	function
1	alpha-enolase isoform X1	ENO1	220	47.3	a structural lens protein (tau-crystallin) in the monomeric form
2	alpha-enolase isoform X3	ENO1	170	47.3	

### Validation of anti-ENO1 autoantibody as biomarker in NSCLC detection

**Figure 2:**
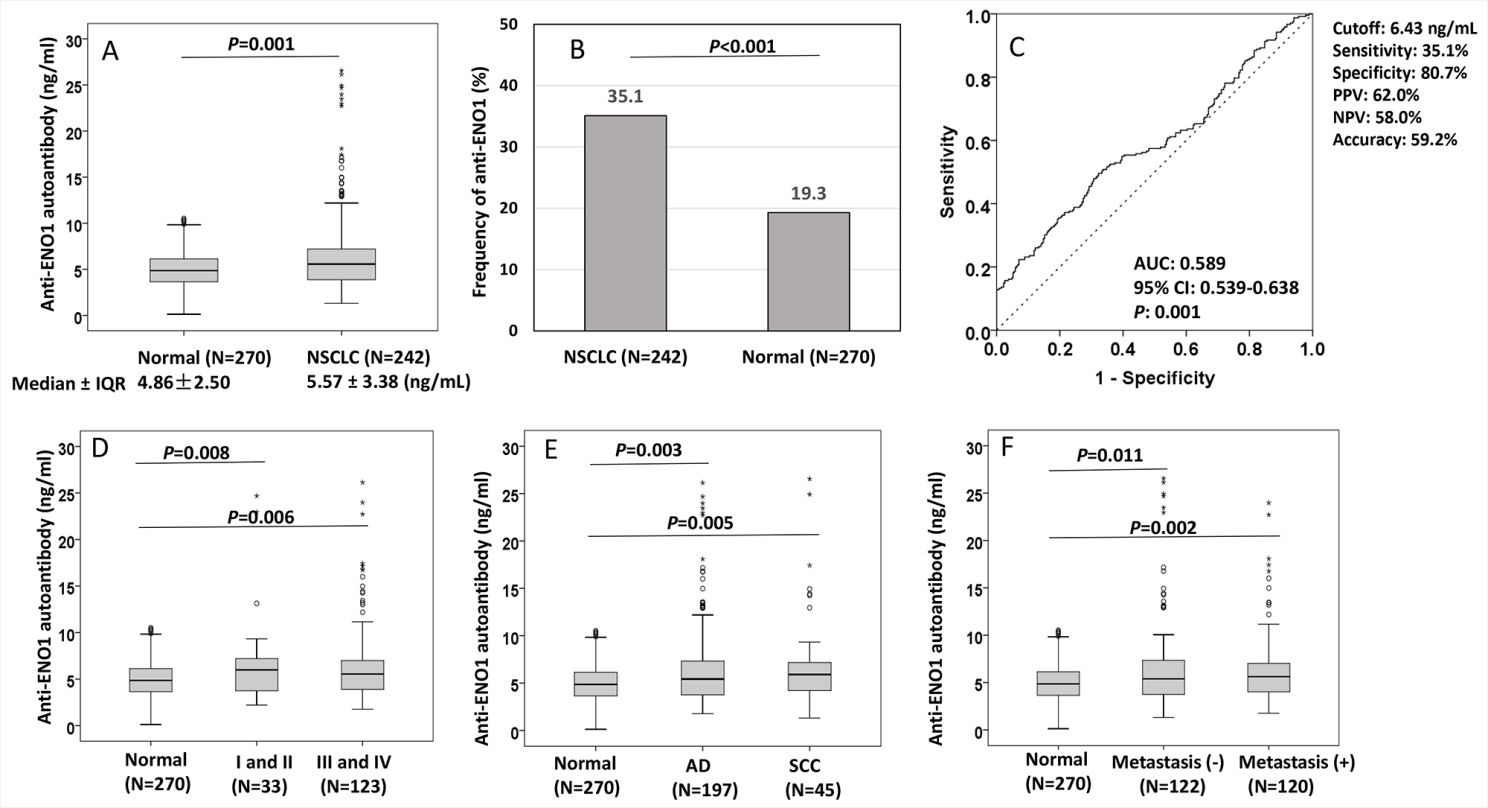
Distribution and ROC analysis of anti-ENO1 antibody in sera from patients with NSCLC and normal control individuals **(A, D-F)** Comparison of autoantibody level of anti-ENO1 in NSCLC patients and subgroups (stage, histology and metastasis) compared to normal individuals. Box and Whisker plots for serum level of anti-ENO1 in patients and normal individuals. The line within the box marks the median, and the 25^th^ and 75^th^ percentiles are presented by the edges of the area, which is known as inter-quartile range (IQR). The bars indicate 1.5 times of the IQR from upper or lower percentiles. *P*: Mann-Whitney test. **(B)** Frequency of anti-ENO1 autoantibody in NSCLC patients and normal individuals. *P*: χ^2^ test. (C): ROC curve of autoantibody to ENO1.

To validate the potential of autoantibody against ENO1 as biomarker in NSCLC detection, the purified recombinant ENO1 protein was used as coating antigen in quantitative ELISA for detection of anti-ENO1 concentration in sera from 242 patients with NSCLC and 270 normal individuals in a validation set of samples. The cutoff value of anti-ENO1 (6.43 ng/ml) designating positive reaction was established as the optimal Youden's Index with regarding to ROC analysis. The autoantibody level of anti-ENO1 was significantly higher in sera from NSCLC patients (median ± IQR: 5.57 ± 3.38 ng/ml) than that in normal individuals (median ± IQR: 4.86 ± 2.50 ng/ml) (*P*=0.001, Figure 2A). The frequency of anti-ENO1 in sera form patients with NSCLC (35.1%) was higher than that in normal individuals (19.3%) (*P*<0.001, Figure 2B). ROC analysis showed that anti-ENO1 can differentiate NSCLC patients from normal individuals with AUC (95%CI) of 0.589 (0.539-0.638), sensitivity of 35.1%, specificity of 80.7%, positive predict value (PPV) of 62.0% and negative predictive value (NPV) of 58.0% (Figure 2C). In the subgroup analysis of autoantibody level, all of the subgroups including stages (*P* value for I+II and III+IV: 0.008 and 0.006, Figure 2D), histological (*P* value for AD and SCC: 0.003 and 0.005, Figure 2E) and metastasis (*P* value for yes or no: 0.002 and 0.011, Figure 2F), were observed to have similar results compared to normal control group. Table 2 shows that the median and frequency of anti-ENO1 antibody were not found to be significantly different in every comparison group (histology: AD vs SCC, stage: I+II *vs* III+IV, metastasis: yes *vs* no, smoking: yes *vs* no, gender: male *vs* female, age: ≤60 y *vs* >60 y). However, the ability in distinguishing NSCLC from normal individuals was found to have statistical significance in stage III+IV (AUC: 0.584, *P*=0.008) and in patients with age >60 y (AUC: 0.626, *P*=0.001), but boundary significance in stage I+II (AUC: 0.596, *P*=0.073) and in age ≤60 y (AUC: 0.562, *P*=0.067) (Table 2).

**Table 2:** Anti-ENO1 level and area under the curve in different characteristics in validation set

	N	median	IQR	P^a^	Frequency (%)	P^b^	AUC	95% CI	P
Histology									
AD	197	5.42	3.63	0.334	69 (35.0)	0.946	0.579	0.526-0.633	0.003
SCC	45	5.91	2.98		16 (35.6)		0.630	0.538-0.721	0.005
Stage									
I and II	33	6.00	3.58	0.786	12 (36.4)	0.881	0.596	0.484-0.707	0.073
III and IV	123	5.55	3.17		43 (35.0)		0.584	0.520-0.647	0.008
Metastasis									
No	122	5.4	3.76	0.799	41 (33.6)	0.618	0.580	0.516-0.645	0.011
Yes	120	5.62	3.02		44 (36.7)		0.598	0.534-0.661	0.002
Smoking									
No	157	5.71	4.11	0.486	57 (36.3)	0.601	0.599	0.541-0.657	0.001
Yes	85	5.19	3.07		28 (32.9)		0.570	0.497-0.643	0.051
Gender									
Male	144	5.38	3.3	0.685	49 (34.0)	0.665	0.580	0.52-0.64	0.007
Female	98	5.75	4.38		36 (36.7)		0.602	0.533-0.671	0.003
Age									
≤ 60 y	138	5.78	3.7	0.390	50 (36.2)	0.677	0.562	0.495-0.629	0.067
> 60 y	104	5.34	3.23		35 (33.7)		0.626	0.552-0.700	0.001

AD: Adenocarcinoma; SCC: Squamous Cell Carcinoma; a: Mann-Whitney test; b: χ^2^ test

In addition, multivariable logistic regression analyses revealed that anti-ENO1 antibody could be used as potential diagnostic biomarker for the identification of patients with NSCLC, AD or SCC after adjustment for age and gender (*P*<0.05 for all) (Table 3).

**Table 3:** Multivariable logistic analyses for anti-ENO1 and various diagnostic factors in NSCLC patients

Comparison	Variables	OR (95%CI)	P
NSCLC vs Normal			
	Age (≤60 y vs >60 y)	1.03 (0.72-1.48)	0.870
	Gender (Female vs Male)	1.09 (0.76-1.56)	0.650
	anti-ENO1 (≥ 6.43 vs < 6.43 ng/ml)	2.27 (1.52-3.40)	<0.001
AD vs Normal			
	Age (≤60 y vs >60 y)	0.97 (0.66-1.42)	0.880
	Gender (Female vs Male)	1.41 (0.96-2.06)	0.080
	anti-ENO1 (≥ 6.43 vs < 6.43 ng/ml)	2.27 (1.49-3.46)	<0.001
SCC vs Normal			
	Age (≤60 y vs >60 y)	1.31 (0.68-2.53)	0.420
	Gender (Female vs Male)	0.25 (0.10-0.63)	0.003
	anti-ENO1 (≥ 6.43 vs < 6.43 ng/ml)	2.39 (1.19-4.81)	0.015

AD: Adenocarcinoma; SCC: Squamous Cell Carcinoma

### Combinational use anti-ENO1 antibody and protein biomarkers (CEA and CYFRA 21-1) can improve sensitivity in diagnosis of lung cancer

**Figure 3:**
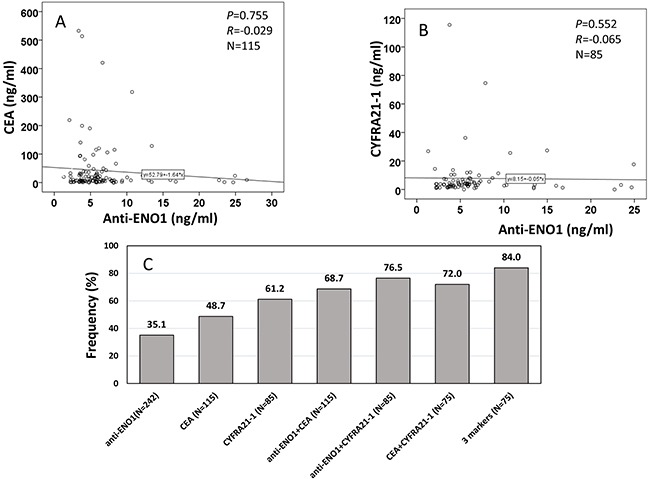
Correlation of anti-ENO1 level with CEA, and CYFRA 21-1 in sera from patients with NSCLC **(A and B)** show autoantibody level of anti-ENO1 has no correlation with CEA (A) and CYFRA 21-1 (B) in NSCLC patients. *P*: Spearman's test, *R*: Correlation coefficient in Spearman's test. **(C)**: frequency/sensitivity of anti-ENO1, CEA, CYFRA 21-1 and their combination in NSCLC patients.

There have been many reports concerning the use of CEA and CYFRA 21-1 in LC detection [16–20], and the results suggested that these markers may be useful in diagnosing LC [16–18, 20, 21]. Of 242 NSCLC patients, information of CEA level was available for 115 patients (median ± IQR: 9.90 ± 28.64 ng/ml), and CYFRA 21-1 level was available for 85 patients (median ± IQR: 3.75 ± 4.46 ng/ml). The detection of serum CEA and CYFRA 21-1 level were carried out by using the Electo-chemiluminescence immunoassay (ECLIA) kit followed the manufacturer's manual (Roche, USA). The threshold values for CEA and CYFRA 21-1 were setup at ≥10 ng/ml [22] and 3.3 ng/ml [23]. Firstly, we investigated the correlation of anti-ENO1 level with CEA (*R*=-0.029, *P*=0.755) or CYFRA 21-1 (*R*=-0.065, *P*=0.552) level and did not found statistical significance (Figure 3A and 3B), which suggests that anti-ENO1 and CEA or CYFRA 21-1 are independent biomarkers in patients sera. The positive frequency in NSCLC patients for each single biomarker was 35.1%, 48.7% and 61.2% for anti-ENO1, CEA and CYFRA 21-1, respectively (Figure 3C). When we combined anti-ENO1 with CEA or CYFRA 21-1, the frequency increased to 68.7% and 76.5%, respectively. The frequency reached at 84.0% when combined detection of anti-ENO1, CEA and CYFRA 21-1 was used (Figure 3C).

## DISCUSSION

In order to identify specific LC biomarker, we initially screened 20 serum samples from patients with NSCLC and 20 normal individuals in a discovery set, and found that 20% of the patient sera contained autoantibodies against a cellular protein with molecular weight around 47 KDa in Western blotting analysis. Therefore, an immunoproteomic approach was used to identify this protein and found this 2DE-Western blotting-positive spot corresponded to ENO1. This approach was named as SERPA and successfully applied to identify and character many kinds of autoantigens in various cancers, such as eukaryotic elongation factor 2 (EEF2), enolase1(ENO1), aldolase A (ALDOA), glyceraldehyde-3-phosphate dehydrogenase (GAPDH) and heterogeneous nuclear ribonucleoproteins (HNRNP A2B1) in Melanoma [24], peroxiredoxin 6 in esophageal cancer, triophosphatase isomerase (Tim) [25], superoxide dismutase (MnSOD) in lung cancer [26], RS/DJ-1 in breast cancer [27]. In our previous study, alpha-enolase was identified as an autoantigen in liver fibrosis by using this approach [14]. Although SERPA has some drawbacks associated with limits of 2-DE, it remains a very robust method for evaluation of the humoral response to cancer.

Enolase was originally characterized as an enzyme involved in glycolytic metabolism. There are three isoforms in mammalian cells: ENO1 (α), ENO2 (β) and ENO3 (γ). ENO1 was found to exist on the cell surface functioning as one of the plasminogen receptors [28], and was responsible for NSCLC proliferation and metastasis through FAK-mediated PI3K/AKT pathway [29]. Chang et al identified ENO1 as LC associated antigen using similar serological approach and showed that increasing expression of ENO1 is a prevailing phenomenon in patients with NSCLC and its expression status is tightly correlated with disease recurrence and survival [30]. The autoimmune responses to ENO1 have been previously described in cancer patients and it has been demonstrated that ENO1 autoantibody may serve as a prognostic marker to monitor disease progression of these patients [31, 32]. Our previous study indicated that the presence of autoantibody against ENO1 may be a predictive marker for better prognosis of liver diseases [14]. To validate our proteomic results, anti-ENO1 antibody was examined in sera from 242 NSCLC patients and 270 normal controls. It was showed that the level and frequency of anti-ENO1 in sera from patients with NSCLC are significantly higher than that in sera from normal individuals, and detection of anti-ENO1 could differentiate NSCLC from normal individuals with AUC (95%CI) of 0.589 (0.539-0.638). There is no significant difference in frequency of anti-ENO1 in different stage, histology or metastasis status of NSCLC. This may suggest that the appearance of anti-ENO1 may have association with the development of NSCLC, but may have less association with the progression of NSCLC, and likely indicate that the antigen identified early- as well as late-stage disease.

Potential noninvasive lung cancer biomarkers in biological fluids have recently been discovered [33]. To date, a variety of NSCLC biomarkers have been identified and the most extensively studied circulating protein markers include CEA, CYFRA 21-1, NSE and CA-125 [16, 18, 19, 34, 35]. Because their sensitivity and specificity are far from satisfactory, their clinical applicability is limited and they have not been generally recommended as a tool for the early detection of LC [36]. Doseeva et al recently confirmed the value if using a mixed panel of tumor antigens (CEA, CA-125, and CYFRA 21-1) and one autoantibody (NY-ESO-1) in the early detection of NSCLC in high-risk individuals, and they found the 4-biomarker panel was able to discriminate NSCLC cases from controls with 74% sensitivity, 80% specificity, and 0.81 AUC in the training set and with 77% sensitivity, 80% specificity, and 0.85 AUC in the independent validation set [37]. In the present study, we combined detection of autoantibodies against ENO1, CEA and CYFRA 21-1, and found it could enhance sensitivity for the diagnosis of NSCLC. These results suggest that autoantibody against ENO1 could potentially act as a complementary clinical biomarker with tumor proteins, such as CEA and CYFRA 21-1, for serological detection of NSCLC.

In conclusion, in the present study, we identified ENO1 as autoantigen using SERPA. ENO1 can elite humoral immune response in NSCLC and have association with the tumorigenesis of NSCLC. Furthermore, autoantibody against ENO1 could be a potential diagnostic biomarker and improve the sensitivity of CEA and CYFRA 21-1 in the diagnosis of NSCLC. Further large-scale validation studies will be needed to determine the sensitivity, specificity and positive predictive value of this marker in real-world screening scenarios.

## MATERIALS AND METHODS

### Serum samples

Two independent sample sets (discovery set and validation set) were used in this study. The discovery set including 20 patients with NSCLC at stage I and 20 normal individuals as control matched by age, gender and smoking were collected from the New York University (NYU) Lung Cancer Biomarker Center, a member of the National Cancer Institute-sponsored Early Detection Research Network (NCI-EDRN). Blood samples from 242 patients with NSCLC in validation set were obtained from the First Affiliated Hospital of Zhengzhou University between March 2013 and April 2014. Control serum samples from 270 healthy individuals in validation set, matched to patients by age and gender, were selected from a census of angiocardiopathy diseases carried out in Zhengzhou City. All patients have been newly pathological diagnosed as primary NSCLC and have not received radiotherapy or chemotherapy before sample collection. All of the control participants had no evidence of cancer history and lung diseases. The characteristics of patients and controls are shown in Table 4. All subjects included in the study provided written informed consent. The study protocol was approved by the Medical Ethics Committee of Zhengzhou University (Zhengzhou, China).

**Table 4:** Characteristics of participants

	Discovery set		Validation set	
Group	NSCLC (N=20)	Normal (N=20)	NSCLC (N=242)	Normal (N=270)
Age, mean ± SD (range)	63.6 ± 4.5 (53-75)	64.5 ± 5.3 (55-78)	58.5 ± 10.5 (27-84)	58.5 ±10.7 (31-84)
≤ 60 y	4 (20.0)	5 (25.0)	138 (57.0)	153 (56.7)
>60 y	16 (80.0)	15 (75.0)	104 (43.0)	117 (43.3)
Gender				
Male	6 (30.0)	6 (30.0)	144 (59.5)	166 (61.5)
Female	14 (70.0)	14 (70.0)	98 (40.5)	104 (38.5)
Smoking				
No	0 (0.0)	0 (0.0)	157 (64.9)	168 (62.2)
Yes	20 (100.0)	20 (100.0)	85 (35.1)	102 (37.8)
Histology				
AD	18 (90.0)		197 (81.4)	
SCC	2 (10.0)		45 (18.6)	
Stage				
I	20 (100.0)		20 (8.3)	
II	0		13 (5.4)	
III	0		29 (12.0)	
IV	0		94 (38.8)	
unknown	0		86 (35.5)	
Metastasis				
No	16 (80.0)		122 (50.4)	
Yes	4 (20.0)		120 (49.6)	

AD: Adenocarcinoma; SCC: Squamous Cell Carcinoma; SD: standard deviation

### Cell culture

The NSCLC cell line H1299 was purchased from American Type Culture Collection (ATCC, Manassas, VA), and cultured in DMEM (Dulbecco's modified Eagle's medium, Invitrogen, Carlsbad, CA) supplemented with 10% fetal bovine serum (FBS), 100 units/ml penicillin and 100 units/ml streptomycin. Cells grown in 75-cm^2^ Falcon tissue culture flasks were allowed to reach 95% confluence. Then, cells were rinsed once with DMEM without FBS and removed from the flask by incubating them with a solution containing trypsin-EDTA (Gibco, Carlsbad, CA), and harvested in a 15 ml centrifuge tube.

### Two-dimensional gel electrophoresis (2-DE) analysis

Briefly, total protein extractions of H1299 cells was directly lysed in rehydration sample buffer (8 M Urea, 50 mM dithiothreitol (DTT), 4% 3-[(3-cholamidopropyl) dimethylammonio] -1-propanesulfonate (CHAPS), 0.2% carrier ampholytes) as provided by Bio-Rad Laboratories (Hercules, CA) and were vortexed vigorously for 1 h at room temperature (RT). Insoluble substances were removed by centrifuge at 16,000 × g for 30 min at 4°C. Supernatant was collected and protein concentration was measured by the Bradford assay (Bio-Rad, Hercules, CA). A total of 200 μg protein was applied on a pH 3–10, 11-cm isoelectric focusing (IEF) strip (Bio-Rad, Hercules, CA). IEF was performed at a current of 50 mA per gel, 300 V for 30 min, followed by 3,500 V for 2.5 h, and additional 8,000 V for 5 h. Strips were immediately stored at −80°C for the second dimensional gel electrophoresis (2-DE) analysis. For the second dimensional electrophoresis, 10% SDS-polyacrylamide gels (SDS-PAGE) were used. Proteins were transferred onto nitrocellulose membrane (Osmonics Inc., MA) for subsequent Western blotting analysis or stained with 0.1% Coomassie blue R-250 prepared in 40% methanol/10% acetic acid. The spots were visualized using PDQuest 2-DE analysis software as described in the manufacturer's manual (Bio-Rad, Hercules, CA).

### One- and two-dimensional western blotting and proteomic analysis

In order to screen the autoantibody-positive sera, H1299 cells were lysed directly in Laemmli's sample buffer and loaded onto 10% SDS-PAGE gel, which is then transferred onto nitrocellulose membrane (Osmonics Inc., MA) for Western blotting. The membrane was then cut into 0.5-cm wide stripes. After blocking with 5% nonfat milk prepared in Tris-buffered saline (TBS), containing 0.05% Tween-20 (TBST), for 1h at RT, the nitrocellulose membrane strips were incubated with sera at a dilution of 1:200. Horseradish peroxidase-conjugated goat anti-human IgG (Caltag Laboratories, San Francisco, CA) was used as secondary antibody with a dilution of 1:10,000 for 1h at RT. The positive bands were detected with Enhanced Chemiluminescence (ECL) kit (Amersham, Arlington Heights, IL). For 2-DE Western blotting, the proteins on 2-DE gel are directly transferred onto nitrocellulose membrane and incubated with two pools of five sera from patients with NSCLC and five normal individuals in the discovery set at a dilution of 1:500.

### Mass spectrometry analysis

After identifying the interesting protein spots, protein spots from the 2-DE reference gel were excised and digested to perform liquid chromategraphytandem mass spectrometry (LC-MS/MS) analysis. MS/MS spectra derived from peptides were submitted for database search using TurboSequest (available in Bioworks version 3.3.1) against the human IPI database (v3.48), in both correct and reverse orientations to enable false-discovery rate (FDR) calculation. The following filters were applied in Bioworks: DCn ≥ 0.85; consensus score ≥ 10.0; protein probability ≤ 1 × 10^−3^; and X_corr_ ≥ 1.5, 2.0 and 2.5, for singly-, doubly- and triply charged peptides, respectively.

### Expression and purification of ENO1 recombinant protein

cDNA encoding human ENO1 was amplified by PCR from a commercial plasmid PMD18T-ENO1 (Sino Biological Inc., Beijing, China). For the expression and purification of recombinant ENO1 protein, the full length ENO1 cDNA was subcloned into expression vector pET-30a which was designed to produce a fusion protein with N-terminal 6× histidine and T7 epitope tags. Recombinant ENO1 protein was further expressed in *E.coli* BL21 (DE3) cells and purified using nickel column chromatography. The protocol used for high-level expression and purification of 6× His-tagged proteins were performed as described (QIAGEN Inc., Valencia, CA, USA). Elution buffer (8M urea, 0.1M NaH_2_PO_4_, 0.01M Tris, pH4.5) was used to elute the recombinant protein. The purified recombinant proteins were further analyzed by electrophoresis on SDS-PAGE, and identified by Western blotting using commercial monoclonal anti-ENO1 antibody.

### Autoantibody measurement by quantitative Enzyme-linked immunosorbent assay (ELISA)

Serum IgG-type ENO1 autoantibody was detected by ELISA, and human IgG antigen was used as standard reference. Briefly, ENO1 protein was diluted in coating buffer (50mM sodium carbonate/bicarbonate pH9.6) to a final concentration of 0.5 μg/ml, and human IgG antigen (Beijing Dingguo Changsheng Biotechnology) was diluted in coating buffer to final concentrations of 300, 250, 200, 150, 100, 50, 10 and 0 ng/ml to generate standard curve for each plate. 100 ul diluted antigens were added into each well for coating at 4°C for overnight. After being blocked each well with 2% Bovine Serum Albumin (BSA, Sigma, USA) for overnight at 4°C, the plates were washed three times by PBST. Human serum samples at 1: 200 dilutions were added to the ENO1 coated wells and incubated for 2h at RT followed by washing three times by PBST. Horseradish peroxidase-conjugated goat anti-human IgG (Santa Cruz Biotechnology Inc., Dallas, TX, USA) at 1:10,000 dilution and the substrate 3,3′,5,5′-Tetramethylbenzidine (Sigma-Aldrich, St. Louis, MO, USA) were used as detecting reagents. Finally, 50 ul stopping solution (2M H_2_SO_4_) was added into each well and the optical density (OD) values were obtained by using a microplate reader (Thermo Fisher Scientific) at dual wavelength of 450 and 620 nm. The relative expression of autoantibodies was calculated and adjusted based on the standard curve of each plate. A positive and negative control were set in each plate to ensure the accuracy of the results. Each sample was tested in duplicate and the average value was used for the further analysis. All the ELISA positive serum samples were confirmed by using Western blotting analysis further.

### Statistical analyses

Due to the sera autoantibody against ENO1 was not normally distributed (Shapiro Wilk's test), nonparametric Mann-Whitney U tests were used to compare differences of antibody levels between two groups. χ^2^ tests were used to compare the differences of frequency between two groups. A multivariable logistic regression model was used to calculate odds ratios (ORs) for age- and sex-adjusted cases associated with NSCLC, AD or SCC according to serum anti-ENO1 levels. Spearman's test was used to evaluate the correlation between anti-ENO1 autoantibody level and concentration of carcinoembryonic antigen (CEA) or cytokeratin 19 fragments (CYFRA 21-1). The receiver operating characteristic (ROC) analysis of anti-ENO1 for the distinguishing of NSCLC from controls, leading to estimates of area under the curve (AUC) with 95% confidence interval (CI). The optimal cutoff thresholds for designating positive reaction were determined at the point on the ROC curve at which Youden's index (sensitivity + specificity -1) was maximal. Differences were considered statistically significant when *P* < 0.05. Statistical analyses were performed using SPSS software (version 18.0).

## Abbreviations

AD: adenocarcinoma
ATCC: American Type Culture Collection
AUC: area under the curve
CEA: carcinoembryonic antigen
CHAPS: 3-[(3-cholamidopropyl) dimethylammonio] -1-propanesulfonate
CI: confidence interval
CYFRA 21-1: cytokeratin 19 fragments
DMEM: Dulbecco's modified Eagle's medium
DTT: dithiothreitol
ENO1: alpha-enolase
FBS: fetal bovine serum
FDR: false-discovery rate
IEF: isoelectric focusing
IQR: inter-quartile range
LC: lung cancer
LC-MS/MS: liquid chromategraphytandem mass spectrometry
LDCT: low dose spiral computed tomography
NCI-EDRN: National Cancer Institute-sponsored Early Detection Research Network
NSCLC: non-small cell lung cancer
OD: optical density
OR: odds ratios
PPV: positive predict value
ROC: receiver operating characteristic
RT: room temperature
SCC: squamous cell carcinoma
SDS-PAGE: SDS-polyacrylamide gels
SERPA: serological proteome analysis
TAA: tumor-associated antigen
TBS: Tris-buffered saline
TBST: TBS containing 0.05% Tween-20
2-DE: two-dimensional gel electrophoresis.
